# Virus-Induced T Cell-Mediated Heterologous Immunity and Vaccine Development

**DOI:** 10.3389/fimmu.2020.00513

**Published:** 2020-03-31

**Authors:** Kathrin Balz, Lilith Trassl, Valerie Härtel, Philipp P. Nelson, Chrysanthi Skevaki

**Affiliations:** German Center for Lung Research (DZL), Institute of Laboratory Medicine, Universities of Giessen and Marburg Lung Center (UGMLC), Philipps University Marburg, Marburg, Germany

**Keywords:** cross-protection, immune memory, molecular mimicry, TCR repertoire, T cell epitope, virus-induced immunity, immunopathology, immunomodulation

## Abstract

Heterologous immunity (H.I.) is a consequence of an encounter with a specific antigen, which can alter the subsequent immune response to a different antigen. This can happen at the innate immune system level—often called trained immunity or innate immune memory—and/or at the adaptive immune system level involving T memory cells and antibodies. Viruses may also induce T cell-mediated H.I., which can confer protection or drive immunopathology against other virus subtypes, related or unrelated viruses, other pathogens, auto- or allo-antigens. It is important to understand the underlying mechanisms for the development of antiviral “universal” vaccines and broader T cell responses rather than just subtype-specific antibody responses as in the case of influenza. Furthermore, knowledge about determinants of vaccine-mediated H.I. may inform public health policies and provide suggestions for repurposing existing vaccines. Here, we introduce H.I. and provide an overview of evidence on virus- and antiviral vaccine-induced T cell-mediated cross-reactive responses. We also discuss the factors influencing final clinical outcome of virus-mediated H.I. as well as non-specific beneficial effects of live attenuated antiviral vaccines such as measles and vaccinia. Available epidemiological and mechanistic data have implications both for the development of new vaccines and for personalized vaccinology, which are presented. Finally, we formulate future research priorities and opportunities.

## Introduction

Heterologous immunity (H.I.) arises from previous infections, which alter the immune response to a subsequent infection with a different pathogen (1). This mechanism is more likely to occur between closely related antigens, but may also occur among unrelated antigens, including bacteria, viruses, protozoa, and parasites. H.I. may alter the outcome of infections by providing sufficient immune protection or, in other cases, aggravating immunopathology (2).

**Figure 1:**
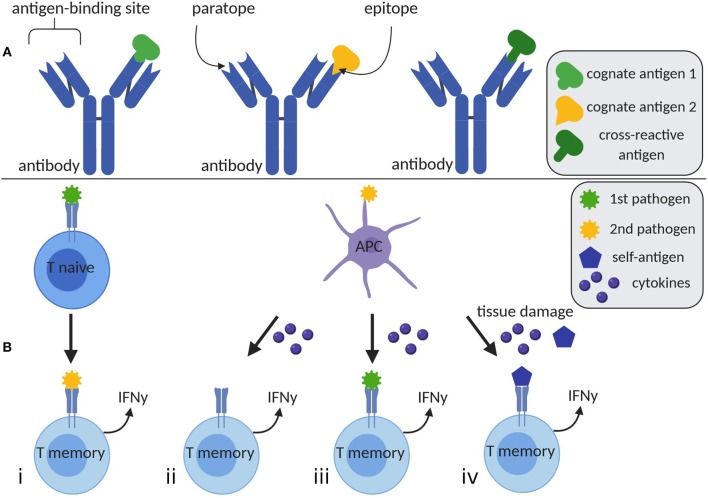
Humoral and cellular mediated heterologous immunity. **(A)** A single antibody has the ability to bind distinct antigens 1 and 2 by different paratopes at the antigen-binding site. Furthermore, it is able to detect a cross-reactive antigen, whose epitope is similar to the one of antigen 1. **(B)** (i) T memory cells may be activated by an unrelated second pathogen, which is cross-reactive with the first encountered pathogen. (ii) The appearance of a second pathogen may elevate cytokine levels, which potentially lead to TCR-independent T cell activation. (iii) Simultaneous presence of cytokines and remaining antigens of previously encountered pathogens may stimulate T cells. (iv) High levels of cytokines and tissue damage due to inflammation or chronic diseases result in increased concentrations of self-antigens, which may be engaged by T cells. Created with BioRender.com, adapted from Welsh et al. (3). APC, antigen presenting cell; IFNγ, interferon γ.

H.I. is mediated by T memory cells or antibodies (Figure 1). Immunoglobulins recognize antigens when antigenic epitopes attach to paratopes (Table 1) at the antigen-binding site. Antibodies are potentially polyspecific, capable of binding different epitopes to various antigens. Furthermore, epitopes sharing similar sequences may bind to the same paratope, providing cross-protection (4). In the context of molecular mimicry, antibodies may also react to self-antigens, eliciting autoreactive immunopathology (5).

**Table 1:** Glossary.

Heterosubtypic	Referring to different serotypes of influenza A virus, which are defined based on the surface proteins hemagglutinin (HA) and neuraminidase (NA).
HLA molecule	The human leucocyte antigen is located on cell surfaces and may present antigenic peptides to T cells.
Immunodominance	Only a few (immunodominant) epitopes are preferentially targeted by the immune response. The remaining epitopes evoke barely detectable T cell responses.
Molecular mimicry	An alignment of pathogenic structures with those of the host, which leads to immune evasion. However, structure similarity of pathogens and self-antigens may elicit autoreactive immune responses.
Paratope	A segment of an antibody's antigen-binding site, which complementarily binds an epitope.
Private specificity of TCR repertoire	TCR repertoires, which are different among individuals.
TCR repertoire	All T cell receptor clonotypes expressed by an organism.

Likewise, cellular-mediated H.I. plays a role in immunomodulation. This may be elicited via T cell receptor (TCR) cross-reactivity (one possible mechanism of H.I.), recognizing similar but distinct antigens or even autoantigens. T cells may also be activated non-specifically by cytokines [reviewed in (3)]. Cross-reactive antigens elicit an expansion of T memory cells, leading to a modified T cell memory pool, a change in patterns of immunodominance, and an altered hierarchy of T cell responses (6). This process heavily depends on the individual private specificities of TCR repertoires and ultimately results in a modified T cell response (7).

Trained immunity, also known as innate immune memory, is a recently described adaptation of innate immune cells following antigenic exposure. Epigenetic reprogramming leads to production of inflammatory mediators and a shift in cellular metabolism, providing an enhanced response to secondary stimulation [reviewed in (8)]. Thus, physiological processes such as mucosal tolerance, restriction of tissue damage, innate immunity maturation, and non-specific vaccine-mediated protection are achieved. Nevertheless, trained immunity can become maladaptive, causing immune paralysis or hyperinflammation [reviewed in (9)].

This review presents recent scientific findings regarding virus- or antiviral vaccine-induced T cell-mediated H.I. and thus provides some background for the discussion on benefits and risks of H.I. Implications for future research priorities for vaccine development are also considered.

## Virus- and Antiviral Vaccine-Induced T Cell-Mediated Heterologous Immunity

### Influenza Virus

Naïve T cells of donors who self-reported as having no influenza A Virus (IAV) H1N1/09 exposure or influenza symptoms can recognize unique strain-specific epitopes using tetramer staining, whereas the same donors' memory T cells recognize conserved epitopes of the surface protein hemagglutinin (HA) (10). In H1N1/09 infected or vaccinated donors, the frequency of naïve T cells recognizing unique epitopes was significantly higher compared to conserved epitope-specific T cells (10). This has also been shown for CD4^+^ (10) and CD8^+^ T cells in mice (11).

Such observations suggest that H.I. influences the severity of infection (10). An age-related dampening of T-cell mediated H.I. was observed following a second heterologous infection in ferrets, which allowed the development of significant morbidity (12). These findings are in agreement with other studies focusing on aged animals, which showed that the clinical severity of primary infection is only moderately accentuated (13–16), while heterologous secondary infection induced severe disease (12, 17, 18). The induction of influenza virus-specific memory T cells is extensively investigated as they are responsible for heterologous protection in secondary natural infections with another influenza strain [reviewed in (19)]. Tissue resident memory T cells (T_rm_) in the lung are particularly important in that respect as they are crucial for achieving optimal protection [reviewed in (19)]. Previous animal studies showed that a single intranasal live attenuated IAV vaccine application can evoke long-lasting protection to heterosubtypic challenge via T_rm_ response in the lung with a similar phenotype to those of infected mice (20). Several *in silico* approaches are available to identify T cell immunogenic regions on virus proteins. It has been demonstrated that epitope-rich regions within the nucleoprotein (NP) of the influenza virus contain highly conserved epitopes and therefore present promising targets for a T cell-mediated vaccine due to cross-reactivity with distinct strains (21). Gutiérrez et al. developed a computational method to compare the efficacy of conserved T cell epitopes (EpiCC), which may complement current methods for selecting the best composition of an associated vaccine (22). Furthermore, CD8^+^ T cells recognizing different NP variants were associated with cross-reactive TCR clonotypes against distinct strains (23). This was shown for the immunodominant and abundant human epitopes NP_338−346_ and NP_44−52_ (23). A structural analysis of the associated HLA molecules revealed adoption of similar conformation as a basis for cross-recognition (23).

Spleen cells from IAV-infected animals showed enhanced IFNγ production after *ex vivo* stimulation with the hepatitis C virus (HCV) derived peptide NS3_1073_ (24). Such findings suggest a private repertoire of pre-existing memory T cells, which are reactivated after HCV infection (25). Cross-reactivity was also demonstrated in human peripheral blood mononuclear cells (PBMCs) of HCV positive patients with severe disease which responded to the IAV-specific peptide NA_231−239_ (25). Additionally, PBMCs of hepatitis B virus patients were incubated with Epstein-Barr virus EBV-BMLF1_280−288_ and IAV-M1_58−66_ labeled tetramers and subsequently stained for TCR clones (26). The TCR repertoire of cross-reactive T cells recognizing IAV and EBV epitopes was broader compared to non-cross-reactive T cells and varied among individuals, further supporting an underlying private specificity (26). The concept of H.I. has recently been expanded to include allergens, following demonstration of IAV-mediated protection against allergen-induced experimental asthma (mediated by memory T cells) in a murine model (27).

### Flaviviruses

The high degree of genetic sequence similarity among flaviviruses is known either to have a protective effect or to dampen the elicited secondary immune response [reviewed in (28)]. For Dengue virus (DENV), it is well-known that an infection with one serotype induces strong and long-lasting protective immunity against that specific serotype, whereas a second infection with a heterotypic virus commonly results in severe disease [reviewed in (29)]. Sub-neutralizing antibody concentrations from the first infection facilitate virus entry by promoting Fcγ-receptor uptake, resulting in antibody-dependent enhancement (ADE) of the infection. However, there is increasing evidence of a cross-protective cellular immune response between DENV and Zika virus (ZIKV) [reviewed in (29)]. Memory T cells isolated from DENV seropositive patients recognize both DENV- and ZIKV-associated peptides (30). Furthermore, DENV positive patients responded more strongly to a ZIKV infection compared to DENV negative subjects when assessed using T cell stimulation assays (30, 31). Mouse experiments have also shown, that DENV-exposed pregnant animals were protected against subsequent maternal and fetal ZIKV infection (32). This protection was conferred by CD8^+^ T cells, limiting trans-placental transmission of ZIKV (32). Although cross-reactivity between DENV and ZIKV is the most prominent example, other flaviviruses, such as yellow fever virus (YFV) and Japanese encephalitis virus, also prime T cell responses toward a subsequent heterologous DENV infection in mice (33). In this context, the investigators identified homologous sequences between the flavivirus polyproteins. Peptides derived from the aforementioned sequences were used to prime antigen presenting cells, which were subsequently used to stimulate splenocytes of DENV immunized mice. Some of these peptides induced enrichment of T memory cells as well as IFNγ production and proliferation, confirming cross-reactivity (33).

### Human Immunodeficiency Virus

Human immunodeficiency virus 1 (HIV-1)-specific CD8^+^ T cell clones showed cross-reactivity against some of the other investigated HIV-1 epitopes (34). Additionally, three HIV-1-specific T cell clones recognized the A^*^02 restricted IAV matrix epitope GILGFVFTL (34). Furthermore, a sequence similarity between the known HIV-1 epitope HIV-Gag [SLYNTVATL [HIV-SL9]] and the HCV epitope HCV-NS5b [ALYDVVSKL [HCV-AL9]] has been observed. HIV-SL9 specific T cells of HIV-1 patients, who were not co-infected with HCV, recognized the aforementioned HCV epitope and responded with IFNγ production and expansion (35).

### Hepatitis C Virus

Cross-genotype protective immunity against HCV was first described in 2003 by Lanford et al. who showed that chimpanzees, which recovered from a genotype 1 infection, were subsequently protected from infection with other genotypes (including genotype 4 and combinations of genotypes 1–4). These genotypes express proteins of up to 30% amino acid variance (36). This finding, however, has been challenged by other investigators who showed that chimpanzees developed chronic disease after being re-challenged with other genotypes (37).

CD8^+^ T cell cross-reactivity to NS3 epitopes of two different genotypes (1 and 3) was observed in a study with 53 anti-HCV positive injection drug users. Interestingly, CD8^+^ T cells recognizing both genotypes were more frequent among HCV RNA negative patients than in those with detectable viremia, implying that CD8^+^ T cell-mediated cross-reactivity may protect against chronic infection (38).

In another study, an HLA-restricted epitope (HCV NS3-1406) and its naturally occurring variants from different genotypes showed that the frequency of cross-reactivity between variants as well as their T cell priming capacities varied, depending on the genotype pair (39). Fytili et al. performed a similar study for another dominant HLA-dependent HCV CD8^+^ T cell epitope (HCV NS3-1073), which was associated with clearance of acute infection, and detected cross-reactivity between the genotype 1 variant and variants of genotypes 4, 5, and 6 but not 2 and 3 (40). The level of cross-reactivity observed in this study could be predicted through *in silico* analyses of peptide-MHC complexes and TCR-interacting surfaces based on topology and electrostatic features (41).

The same dominant T cell epitope (HCV NS3-1073) was also found to induce immune response in approximately a third of >100 seronegative individuals upon *ex vivo* stimulation. The presence of CD8^+^ T cells specific for that epitope was attributed to cross-reactivity with epitopes derived from other pathogens. These cells not only reacted to different genotype variants of that epitope but also to epitopes with little sequence similarity of other, unrelated viruses (cytomegalovirus, IAV, EBV) (42). Immunization with a recombinant adenovirus vector containing mycobacteria, Ebola and HIV antigens also led to T cell responses against HCV alongside the transgenic antigens (43). Cross-reactivity between an HCV and a human herpes virus peptide has also previously been demonstrated (44).

### Other Viruses

Severe hand, foot and mouth disease is caused among others by enterovirus 71. A dominant capsid T cell epitope, which is highly conserved among enteroviruses, was identified and found to yield a cross-reactive, HLA-DR restricted response of human CD4^+^ T cells to the poliovirus variant of this epitope (45). Human RV-specific CD4^+^ T cells were shown to recognize epitopes shared among different RV strains (46). Human circulating RV-specific CD4^+^ T cells recognized conserved RV capsid protein epitopes, and T cell-mediated cross-reactivity between different strains was demonstrated (47). Zhao et al. showed that airway CD4^+^ T memory cells specific for a dominant, conserved epitope (SARS-N353) protect against both SARS- and MERS-CoVs and also against bat CoV in HLA transgenic murine models (48). Hepatitis E virus (HEV)-specific CD4^+^ and CD8^+^ T cell responses against different peptide pools from HEV1 were detected in acute HEV3 patients. A similar response against HEV3- and HEV1-peptide pools was detected in one subjectwith HEV1 infection (49). Finally, H.I. between the arenaviruses lymphocytic choriomeningitis virus and Pichinde virus was demonstrated in murine models and found to be T cell epitope and MHC class dependent (50).

## Discussion

### Protection vs. Immunopathology

Overall, virus-induced H.I. appears to be an important determinant for the final outcome of infections and of a plethora of dysregulated immune responses such as in autoimmunity and allograft rejection. In this context, prior antigenic exposures may boost protective responses [e.g., (27)] or induce immunopathology depending on the balance between antigen load and efficiency of effector T cells, which in turn is influenced by a number of factors. For example, in the case of flaviviruses, it has recently become evident that distinct T cell populations, virus serotypes, sequence, and number of infections, and HLA background all shape the immunodominance pattern (29). Additionally, patterns of T cell cytokine response among patients with a secondary DENV infection were associated with severe (51, 52) or mild dengue (53, 54). Although heterotypic antigens were addressed only in one of these studies (52), such observations may indicate involvement of cross-reactive T cells in the clinical manifestation of DENV infections.

In addition to natural viral infections, antiviral vaccines may also drive T cell-mediated H.I. and have a major impact not only against the vaccine antigens but also on completely unrelated pathogens or other antigens. To date, epidemiological evidence supporting the role of live attenuated vaccines in T cell-mediated H.I. is associated with the measles (55–62), the vaccinia (63–66), and the oral polio vaccine (67–69). These vaccines reduced overall mortality and/or risk for asthma, malignancies, and unrelated infections. Furthermore, they induced changes in the numbers or proportions of T and B cells, which, depending on persistence of effects, may influence differentiation, proliferation or survival of associated cells. Non-specific effects of vaccines have often been found to be sex-specific and influenced by revaccination as well as maternal priming. In this regard, knowledge on the potential of specific T cell epitopes (for any given HLA background) to offer protection or cause pathology is crucial for vaccine design including elimination or inclusion of such peptides.

### Implications for Vaccine Development

The ability to predict the magnitude and mechanism of T cell-mediated H.I. (Figure 1B) is crucial for specific vaccine design but also for decisions on public health and vaccination policies. Structural similarity between T cell epitopes seems to be important for eliciting cross-reactive responses. Nevertheless, seemingly distinct epitopes may also bind to the same TCR and induce H.I. This may be explained by the fact that sequence similarity is also dependent on the presence of biochemically similar amino acid substitutions (70). In the context of developing broadly cross-reactive vaccines against viruses with great antigenic heterogeneity, regions of highly conserved proteins among serotypes may elicit cross-reactive T memory cell responses. This approach along with large scale systematic monitoring of circulating strains, as in the case of influenza (in order to minimize mismatch with vaccine-contained strains) may increase vaccine effectiveness.

Besides their specific effect, it is now known that vaccines may also exert a non-specific influence on the immune system (71). For the diphtheria-tetanus-pertussis and measles vaccines, it was shown that the order of vaccination has an impact on overall morbidity and mortality (72). The concept that the most recently administered vaccine leaves a non-specific immunological imprint until subsequent immunization may guide changes in the recommended order of childhood vaccinations. Such changes could result in beneficial non-specific effects with minor changes of existing national vaccination schemes. Similarly, age at the time of (initial or booster) immunization with each existing vaccine may need to be reconsidered based on the accumulating knowledge on immunosenescence and effects of age on virus-induced H.I. Accordingly, time of vaccination has been linked to differences in T cell populations and strength and type of heterologous immune response (73, 74). Sex-specific differences in terms of protective non-specific effects of vaccines such as measles and vaccinia (64, 75–80) have also been described. Modification of vaccine composition (e.g., enrichment of particular proteins or epitopes) or conditions of administration (e.g., age, dose, number of immunizations) could potentially help us achieve the beneficial heterologous effects of vaccines without compromising their primary protective effects (vaccine specific). Indeed, adequate application of knowledge regarding vaccine-mediated H.I. brings us a step closer to precision medicine and personalized vaccinology. Administration of live attenuated vaccines to women as part of preconception health counseling is another measure, which could enhance protection of offspring in the first months of life.

The potential of virus- and antiviral vaccine-induced immunomodulation may also be exploited for novel applications such as preventing infections among elderly and immunocompromised populations or non-infectious inflammatory diseases. In this respect, the choice of a particular adjuvant or pharmacological modulator is also important since these may polarize T cell immune responses toward a specific cytokine output depending on the desired outcome, e.g., induction of T1 type of response for prevention of infection as well as allergies.

### Future Research Priorities

The need for new vaccines with higher efficacy and broader and longer-lasting protection is driven by the moderate protection provided by current seasonal influenza vaccines against the included strains, zoonotic and pandemic influenza threats, and the challenge of complying with annual vaccinations. Several approaches are currently being investigated with varying results and distance from truly universal vaccines. The use of adjuvants, addition of neuraminidase, and inclusion of specific strains induce broader reactive immune responses albeit within the same virus subtype. Additionally, immunogenic influenza HA-stem constructs induce B cells which produce cross-protective antibodies, at least within a group of viruses. A particular promising approach for the development of truly universal influenza vaccines seems to be the induction of T cells reactive to internal viral proteins, primarily of T_rm_ in the respiratory mucosa for timely control of viral replication. Such approaches could also prove useful for developing vaccines against other respiratory viruses such as rhinoviruses. Similarly, knowledge gained from current studies of T cell responses against DENV/ZIKV infections at several time points, and with different clinical presentations and history of infection may inform strategies for developing pan-flavivirus vaccines. Indeed, there is already evidence for cross-reactive immunogenic epitopes contained in these viruses.

Properties of virus-induced H.I. may be leveraged beyond infection protection. We have previously shown an influenza virus-mediated protection over development of experimental asthma in a murine model. The protection was conferred by CD4^+^ and CD8^+^ T memory cells, which were transferred from animals previously infected with influenza or immunized with cross-reactive influenza peptides to sensitized mice before challenge with an allergen. Given the global prevalence of allergies, peptide immunization strategies early in life could potentially induce protective cellular immune responses against viruses and allergen-induced asthma, and complement existing vaccination schedules. Importantly, directing non-specific beneficial effects of existing live attenuated viral vaccines against other inflammatory disorders including cardiovascular disease and cancer could be a quantum leap in the fight against non-communicable diseases (65, 81–84).

Further immunological and clinical studies are needed to decipher vaccine-induced H.I.-mediated mechanisms and impact on morbidity and mortality contributing to health promotion. Associated potentiators such as booster vaccinations and maternal priming need to be examined carefully in different socioeconomic settings and with a sex-differential analysis (85).

## Author Contributions

CS and PN planned, structured, and edited the manuscript. PN searched the literature and integrated all contributions. All authors wrote distinct parts of the manuscript and critically read, reviewed, and approved the final version of the manuscript.

### Conflict of Interest

For CS: Consultancy and research funding, Hycor Biomedical and Thermo Fisher Scientific; Consultancy, Bencard Allergie; Research Funding, Mead Johnson Nutrition (MJN). The remaining authors declare that the research was conducted in the absence of any commercial or financial relationships that could be construed as a potential conflict of interest.
